# Removal of arsenic as a potentially toxic element from drinking water by filtration: A mini review of nanofiltration and reverse osmosis techniques

**DOI:** 10.1016/j.heliyon.2023.e14246

**Published:** 2023-03-04

**Authors:** Hoda Pezeshki, Majid Hashemi, Saeed Rajabi

**Affiliations:** a Department of Environmental Health Engineering, Faculty of Public Health, Kerman University of Medical Sciences, Kerman, Iran; b Environmental Health Engineering Research Center, Kerman University of Medical Sciences, Kerman, Iran; c Student Research Committee, School of Health, Shiraz University of Medical Sciences, Shiraz, Iran; d Department of Environmental Health Engineering, School of Health, Shiraz University of Medical Sciences, Shiraz, Iran

**Keywords:** Heavy metals, Potable water, Nanofiltration, Removal technology, Reverse osmosis

## Abstract

Arsenic is a priority contaminant that enters drinking water through both natural and man-made processes, posing a risk to human health and leading to the development of a variety of illnesses. Since millions of people are exposed to drinking water with a concentration of this pollution that is higher than allowed levels, its removal has become a crucial issue, and this removal is accomplished using a variety of techniques. In this study, the removal of arsenic using two membrane processes-nanofiltration (NF) and reverse osmosis (RO) has been specially investigated in light of the outstanding removal efficiency of arsenic through membrane processes. Arsenic in drinking water must be removed using the right techniques to comply with world health organization (WHO) guidelines. According to the findings of several studies, NF membranes can remove significant amounts of heavy metals, such as arsenic, at low pressures while still producing high-quality water, which lowers operating costs. RO membranes are regarded as yet another efficient membrane technology for eliminating both types of arsenic throughout a wide pH and pressure range. Although the likelihood of membrane clogging can be considered as a restriction in these processes, given the possibility of its modification through the use of proper pre-treatment and also taking into consideration benefits such as the lack of need for chemicals, the absence of sludge production, removal effectiveness up to the WHO standard limit, and the removal of a wide variety of contaminants, they are preferred compared to other techniques in as much as they have the potential to become the most effective method of removal.

## 1 Introduction

Water, a special component of creation and the source of life, has been so integral to human existence for a very long time that it is difficult to even conceive life continuing without it. However, the same water that gives life to humans may also kill them, and this is mostly due to their careless intervention [1,2]. As the rate of global population growth, scientific advancements, and ensuing growth in industrial and agricultural activities increase, water pollution has become one of the most significant worldwide concerns. At the same time, however, freshwater resources have decreased, posing a major threat to public health [[3], [4], [5], [6], [7]]. More than 150 million people worldwide are at risk due to drinking water contamination, which also claims the lives of more than 20 million people each year [3,8].

The term "water pollution" refers to the process of introducing undesirable elements into the water and altering its quality in a way that is damaging to both humans and the environment [9]. Pollution is caused by a variety of factors including climate change, air pollution, reservoir leaks, untreated sewage discharge, population increase followed by overuse of surface and subterranean water resources, mining, industrialization, roadways, and industries. Water supplies are subjected to a variety of contaminants, including pesticides, detergents, phenolic compounds, organic dyes, trihalomethanes, radioactive materials, and heavy metals, which can alter the water's quality and make it unusable as a source of water [3,5,[9], [10], [11], [12]].

Potentially toxic elements are the most dangerous of the water contaminants, and their environmental pollution has become a global issue since their entry into the biological cycle causes fundamental changes in natural and human ecosystems [13,14]. Typically, substances referred to be heavy metals have atomic numbers greater than 20 and densities greater than 5 g/cm^3^ [15]. Some of these metals are necessary for the body to operate normally, but when they enter the body in excessive amounts, both directly and indirectly through the soil, water, and air, they have negative health impacts because of their cumulative nature. So, it's very crucial to keep their quantity under control [16,17]. Although these components are present in the environment naturally, it is recognized that human activity is the principal factor driving up their concentrations in aquatic environments. The quantity of heavy metals entering aquatic ecosystems has grown over the past ten years, and around 40% of lakes now have heavy metal contamination [5]. There are 35 toxic metals that people are exposed to, and 23 of them are heavy metals, with arsenic (As) being one of the most significant [13].

## 2 Arsenic in water

Arsenic is a very poisonous metalloid with the atomic number 33 that exists in both organic and inorganic forms, with the hazards of the inorganic form, which enters the body mostly by drinking water, being significantly larger than the dangers of the organic form [14,15,18]. Arsenic occurs naturally in the oxidation states of arsenate (As^5+^), arsenite (As^3+^), elemental arsenic (As^0^), and arsine (As^3−^), of which arsenite and arsenate are the dominant species of groundwater and surface water, respectively [19]. Natural processes including volcanic eruptions, weathering of arsenic-containing rocks, geothermal water discharge, and biological activity, as well as human activities like quarrying, metal smelting, fuel combustion from fossil resources, and the use of fungicides, herbicides, and wood preservatives, can all cause the environment and drinking water to become contaminated with this heavy metal [15,[20], [21], [22]].

Arsenic is a group I carcinogen heavy metal that has been classified as a priority pollutant in nature. Exposure to it can have a variety of detrimental impacts on health, including disruptions of the nervous, respiratory, and digestive systems. Arsenic contamination of drinking water can lead to many illnesses, including peripheral neuropathy, sleep disorders, impaired memory, chronic cough, lung ailments, gastroenteritis, and dyspepsia [23]. The long-term effects of consuming water with a high concentration of arsenic include skin problems including pigmentation and keratosis, developmental problems, pregnancy abnormalities, diabetes, as well as cutaneous, lung, liver, renal, and bladder cancer [15,[24], [25], [26], [27], [28]]. It has been established that a high percentage of arsenic in drinking water is associated with cardiovascular conditions including Blackfoot disease. Furthermore, studies have shown that exposure to arsenic causes human hypertension [15,29]. Additionally, studies have demonstrated an association between congenital heart disease risk and maternal exposure to As in drinking water, even at low levels (0.5–0.9 μg/L) [30]. As a consequence of the health dangers involved, the World Health Organization (WHO) reduced the allowed quantity of arsenic in drinking water from 50 to 10 μg/L. Additionally, the American Environmental Protection Agency (EPA) indicated that the threshold was 10 μg/L [18]. More than 200 million individuals are exposed to water that has an As content greater than standards. More than 105 nations have reported having arsenic in their water [14,15]. Consequently, water purification to eliminate As has become a crucial topic. Table 1 compares the amounts of arsenic in various nations, with Spain having the lowest levels and Latin America having the highest.

**Table 1:** Level of arsenic found in water resources around the world.

Region	Water resource	Arsenic level	Ref.
Bangladesh	Groundwater	50 μg/L<	[28]
India Peninsula-Haryana	Groundwater	50 ppb<	[31]
Iran-Kurdistan-Delbaran	Urban and Rural	70 ppb	[10]
Latin America	Drinking water	10–2000 μg/L	[32]
Pakistan	Groundwater	120 μg/L	[33]
Southwest Bangladesh	Drinking water	13.1–292 μg/L	[34]
Spain	Drinking water	1–118 μg/L	[28]
Turkey-Aksa ray	Drinking water	10–50 μg/L	[35]
USA-Michigan	Drinking water	10–100 μg/L	[36]

Arsenic contamination is widespread across the world's water supplies, as seen in the table above. According to studies done thus far, NF and RO membranes are incredibly effective in removing arsenic from the water in these areas. For instance, five different RO membranes were employed to clean water in Kurdistan, Iran, with an arsenic content of 80 ppb, and in each case, removal effectiveness of more than 80% was attained [10]. In a Bolivian study, RO systems were able to reject arsenic at a rate that was over 99% [37]. China, a nation where the amount of arsenic in the drinking water is worrying, utilized the NF system to remove the substance and recommended it as an effective way to treat arsenic-rich water [38,39].

## 3 Removal methods of arsenic from water

Among the procedures for purifying water, physical, chemical, and biological ones can be mentioned [40]. Arsenic may be removed from water using a variety of techniques (Table 2), including membrane processes, ion exchange, electrolysis, coagulation and flocculation, oxidation, surface adsorption, and lime lightning [12,24,41]. Most of these methods need a pre-treatment step, which often entails an oxidation step to transform arsenite to arsenate since 3-valent arsenic compounds are 10 times more poisonous than 5-valent arsenic compounds and are neutral compounds up to pH = 9 [4,12,14].

**Table 2:** Comparison of arsenic removal efficiency in different processes.

Removal process	Advantages	Disadvantages	Removal efficiency (%)	Ref.
Oxidation	- Low operating cost - Working in a variety of pH ranges	- Low speed - Sludge production	As^5+^: >95	[3]
Surface adsorption	- Low cost required - Ease of use - Production of minimal by-products - Operation without sludge production - Effective removal of 5-valent arsenic	- Low efficiency in the presence of other organic substances and salts - Require pre-and/or post-treatment (oxidation and/or filtration) - Difficulty removing heavy flocs - Difficulty in 3-valent arsenic removal	As^3+^: 30-60 As^5+^: <90	[3,12,[41], [42], [43]]
Chemical precipitation	- Wide application - Simplicity of the process - Extensive removal of heavy metals - Effective removal of 5-valent arsenic	- High sludge production - Slow process speed - Require large amounts of chemicals - Require pre-and/or post-treatment (oxidation and/or filtration) - Difficulty in 3-valent arsenic removal	As^5+^: 95	[43,44]
Dissolved air flotation (DAF)	- Combining the flocculation process with the removal of algae and humic substances with high color concentration and low turbidity	–	Total As: >99	[45]
Ion exchange	- High speed of the process - Less toxic sludge production - A successful method for eliminating 5-valent arsenic	- Hazardous chemical leakage during resin regeneration - Require pre-and/or post-treatment (oxidation and/or filtration) - Inefficient to remove 3-valent arsenic - Influence of unwanted ions on the efficiency - High cost	As^5+^: 95	[3,42,43,46]
Coagulation (Coagulants: Iron (III) chloride, Aluminium sulfate (Alum), and Polyaluminium chloride (PAC))	- Working in a variety of pH ranges	- High cost - Production of arsenic-contaminated sludge - Additional filtration required	FeCl_3_: 91 Alum: 96 PAC: 99.4	[3,45]
Electrocoagulation	- Sustainable technology - Easy maintenance and repair	- High energy consumption - High investment cost - Sludge production - Depending on the kind and quantity of coagulant	As^5+^: >99	[42,44]
Zero-valent Iron nanoparticles (ZVI)	- Accessibility - Low cost - Non-toxic - High reaction speed - High efficiency	- Particles' propensity to accumulate in solutions - The requirement for sophisticated separation techniques	Total As: 98	[18,42]
ZnO nano-photocatalyst	- Environmentally friendly - Ability to reuse - Stability - Economic	- Particles' propensity to accumulate in solutions - The requirement for sophisticated separation techniques	Total As: 98.3	[42,47]
Membrane processes	- Simple operation - No sludge production - No need for chemicals - No effect on taste and smell - High efficiency of arsenate removal - Low energy consumption	- High investment cost - Membrane sediment formation - Influence of unwanted ions on the efficiency	Total As: 96	[3,43,44,48]

The capacity of the coagulation and flocculation method to purify inorganic flows with metal concentrations between 100 and 1000 mg/L is typically regarded as one of this technology's limitations. Coagulation and sedimentation increase sludge stability and can eradicate sludge bacteria, but one of the primary drawbacks of this procedure is that it produces more sludge overall. The use of chemicals and the ensuing substantial rise in operational expenses is a further disadvantage [40]. Chemical precipitation is another effective approach for eliminating heavy metals, although, despite its widespread usage, it necessitates a considerable amount of chemicals. Among its other disadvantages is the formation of sludge and the subsequent imposition of expenses, as well as the presence of wasted chemicals [40,49]. One of the several forms of physical separation techniques, flotation produces excellent results when combined with other purifying techniques. The benefits of this technology are the removal of tiny and light particles, a quick retention duration, and inexpensive costs [40].

Surface adsorption is another technique of water purification that is regarded as effective and has numerous advantages such as simplicity of use, cost reduction, little by-product production, and flexibility. The fundamental drawback of this approach is the dearth of effective low-cost adsorbents. Additionally, the equilibrium of this process takes a long time to reach. Ion exchange is a physicochemical process that, while producing less sludge than chemical precipitation, has a high start-up cost. It has good efficiency and speed to remove ions even in small amounts. The selectivity of this technique and the emission of dangerous compounds during resin regeneration are additional drawbacks [40,42]. Electrochemical treatment is a process that involves applying direct electric current to the electrodes of a chemical cell, and the requirement to use more little chemicals and less generate sludge overcomes the benefits. This approach has limitations because of the high prices of electricity and basic equipment [40].

## 4 Search strategy

The keywords Reverse osmosis, Nanofiltration, Arsenic, phrases Arsenic Removal from Water by Reverse osmosis, Arsenic Removal from Water by Nanofiltration, Arsenic Removal from Water by Nanofiltration, and Reverse osmosis, and their Persian equivalents were searched in the databases Pub Med, Google Scholar, Science Direct, Civilica, SID, and Magiran to identify literature related to the requested review article. More than 40 articles from among the current studies were reviewed and studied, and eventually, the present research was carried out using 25 of the most pertinent articles from the years 2005–2022.

## 5 Filtration processes in arsenic removal from water

**Fig. 1:**
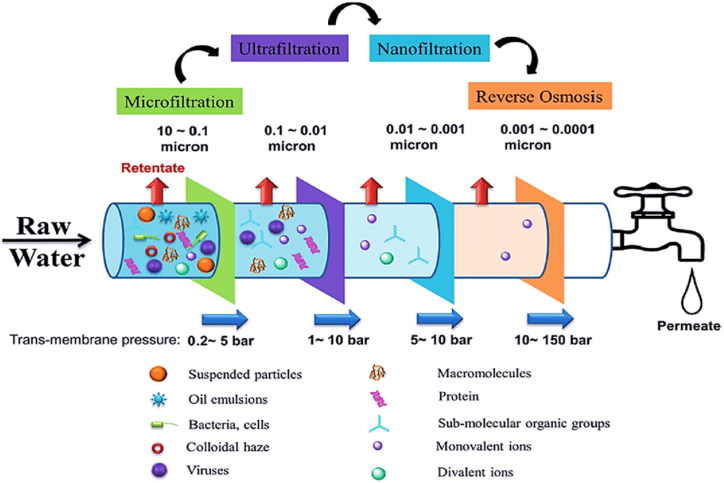
Membrane processes characteristics [52].

The use of membrane processes in water purification has drawn attention recently because of its ease of use, ability to lower prices and the number of operational units, recover valuable products, and expand commercial access to all varieties of membranes globally. These procedures are likewise recognized as the most effective and effective means of removing arsenic [3,8,40]. Membrane processes are non-thermal techniques for purifying a solute-saturated solution. They are classified as low-pressure membranes, which include microfiltration (MF) and ultrafiltration (UF), and pressurized membranes, which include nanofiltration (NF) and reverse osmosis (RO) (Fig. 1) [50]. These procedures are regarded as "clean" technologies in the field of separation since they don't require the use of additives. They are also better than other separation techniques because they are simple to use, highly effective, and don't produce any sludge. Furthermore, they can eliminate a variety of contaminants [3,12]. According to the larger pores of the membranes relative to the size of arsenic particles, microfiltration and ultrafiltration have a low potential for removal and are only efficient for removing large arsenic particles, whereas nanofiltration and reverse osmosis are the most effective procedures for removing arsenic due to having smaller pores [43].

Reverse osmosis and nanofiltration are the most effective filtration techniques. One of the membrane techniques used in water purification is nanofiltration, which is used to remove multivalent ions, organic compounds, viruses, bacteria, and pesticides. For extracting heavy metals from inorganic solutions, this procedure has extremely excellent efficiency. Among its drawbacks is reduced process performance in the event of a rise in pollutant concentrations and filter blockage caused by sediment formed by ions and colloids [11,12,42]. Reverse osmosis is a sophisticated water purification technique that offers a high percentage of ion removal capacity. The advantages of this process include high desalination efficiency, the absence of chemicals, and a reduced need for specialist labor. The drawbacks include membrane clogging, the need to replace them, and an increase in operating expenses, all of which may be avoided by using an appropriate pre-treatment procedure [6,8,12,51].

### 5.1 Nanofiltration

One of the most recent and effective pressure-based membrane techniques for the separation of multivalent ions from monovalent ones uses nanofiltration, which has pores with a size of 0.001–0.01 μm. Its separation properties range between reverse osmosis and ultrafiltration [3,11,42]. Nanofiltration membranes typically have two layers. Protection against system pressure is provided by protective layers, while isolation is accomplished by thin and dense layers [11]. Important polymers that are utilized to create nanofiltration membranes include polyamides, cellulose acetate, cellulose diacetate, cellulose triacetate, piperazine, etc. [53]. These membranes can be found in a variety of shapes, including spiral, sheet, tube, and fiber [11]. High-quality wastewater may be produced using nanofiltration membranes, which can remove a significant portion of heavy metals including arsenic [49]. Reduced consumption expenses, energy expenditures, and operational strain are other benefits of this approach that are important [3]. In order to remove water hardness ions (calcium and magnesium), these membranes are also a useful and acceptable technique [50]. Nanofiltration membranes such as NF90, NF70, NF45, ES-10, UTC-70, TFC-50, and TFN membranes are some examples of those used to remove arsenic from water [3,26].

### 5.2 Reverse osmosis

**Fig. 2:**
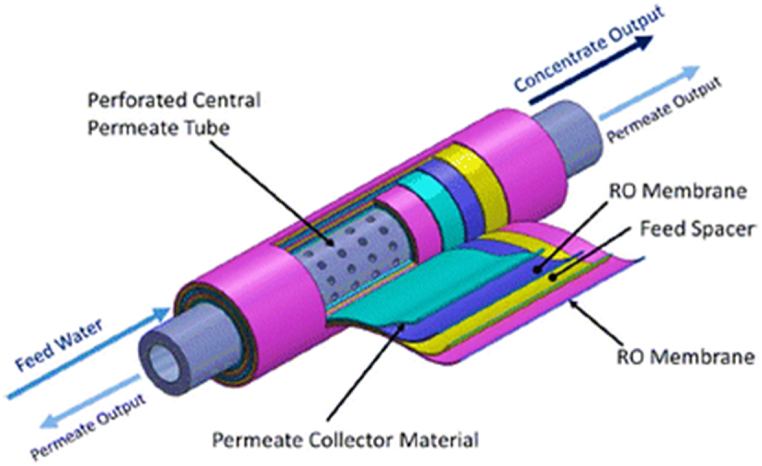
Spiral-wound module of RO membrane [55].

One of the most modern techniques for removing membrane-bound arsenic is reverse osmosis (with a pore size of 0.0001 μm), in which the direction of water through a semi-permeable membrane is turned such that pure water flows from the concentrated side to the dilute side and ions are prevented from passing through the membrane [12,42]. This membrane is capable of filtering out a variety of big molecules, monovalent ions, and extremely tiny contaminants [12,18]. Reverse osmosis membranes, such as nanofiltration, are made of important polymers such as polyamides, cellulose acetate, cellulose diacetate, cellulose triacetate, piperazine, etc. [53]. This technique performs well in a variety of pH and pressure conditions. Other benefits of this technique are its high removal effectiveness, lack of chemical dependence, mechanical toughness, chemical stability, capacity to endure extreme heat, reduced requirement for an experienced operator, and comparatively low energy usage [6,54]. BW30, TFC-ULP, FT30, TFC-SR, PVD, XLE, AD, and BE membranes are some of the reverse osmosis membranes utilized for arsenic removal (Fig. 2) [8,10,26].

The most challenging obstacle to the implementation of membrane processes is the clogging of the membranes produced by numerous impurities such as inorganic compounds, colloidal matter, dissolved organics, and microorganisms, which, if improved, may be predicted to boost performance. Membrane treatments offer significant advantages over water purification and arsenic removal technologies [[56], [57], [58]]. Reverse osmosis and nanofiltration membranes are expensive to invest in initially, and when they clog, sometimes may need to be replaced, which raises the expense [59]. Membrane clogging may be decreased by using low-fouling membrane materials, enhancing system operation conditions, and performing membrane cleaning [58]. The presence of suspended particulates causes clogging to exacerbate and reduces process speed, which may be mitigated by lowering the hardness of the inlet water flowing before filtering and applying proper pre-treatment [12,42]. Arsenate is rejected significantly more in contrast to arsenite, which is neutrally charged since arsenate ions are often negatively charged (HAsO_4_^2−^ and H_2_AsO_4_^−^) in natural water, which is similar to most of the employed NF and RO membranes (H_3_AsO_3_). This is a flaw in this method that can be fixed by raising the pH level that makes arsenite charged [8,10,26,33,42,60]. It should be noted that due to the larger pore size of RO membranes at low pH levels, they can remove a higher percentage of arsenite than NF ones [26].

The lesser removal efficacy of arsenite in comparison to arsenate is another drawback of this approach [42]. Additionally, this method has the disadvantage that nanofiltration membranes, due to their smaller molecular weight, are unable to remove monovalent ions and suffer a significant fall in efficiency as the concentration of pollutants increases [61]. In consideration of the aforementioned, choosing a membrane based on the features and quality of the water would lessen this method's drawbacks and improve the process' effectiveness [12].

### 5.3 Studies of arsenic removal by NF & RO

Arsenic removal by reverse osmosis and nanofiltration has been the subject of several investigations. Akbari et al. evaluated the impact of various parameters on the effectiveness of the nanofiltration process in removing arsenic. The findings of this research demonstrate that the removal rate declines as the concentration of arsenite and arsenate increase because a concentration accumulation layer forms on the membrane's surface and arsenic permeate the membrane. Although the flux of clean water increases as pressure rises, the flux of dissolved materials stays constant, causing dilution and a decrease in the concentration of arsenic, which enhances the removal of arsenate and arsenite. Similarly, increasing the pH enhances the clearance rate by making the arsenic charge more negative. On the other hand, a drop in kinematic viscosity and an increase in permeability are the causes of how much removal is reduced as temperature rises. Since the Donan potential is decreased by the addition of additional salts, the amount of arsenate removed is likewise decreased. For arsenite and arsenate, the greatest efficiency was 95.11% and 99.02% respectively [62].

In a study, Figoli et al. looked into the performance of two nanofiltration membranes (NF30 and NF90) for removing arsenic, and under all test circumstances, the NF90 membrane outperformed the NF30 membrane. This study found that raising pH had a bigger and more substantial impact on the NF30 membrane's ability to remove arsenic than did reducing operating temperature or arsenic concentration [63]. According to Nguyen et al. research, the elimination of arsenite and arsenate increased with increasing pH in the range of 4–10, with arsenite being largely eliminated at pH 8 to 10. Arsenate's removal effectiveness rose from 89 to 96% when the concentration of arsenic was raised from 20 to 100 μg/L, but fell from 44 to 41% for arsenite. Cl^−^ ions increased the efficacy of removing arsenate, while SO_4_^2−^ ions decreased the effectiveness of removing arsenate [64]. According to research by Saitua et al. the NF300 membrane also effectively removed 97% sulfate, 88% hardness, and 75% TDS in addition to 95% arsenate [65].

Malakootian et al. evaluated the effectiveness of the nanofiltration process in removing heavy metals from sulfate-containing waters. Their findings demonstrated that polyamide-type nanofiltration membranes are appropriate for simultaneously removing sulfate and heavy metals from water because they can remove a high percentage of heavy metals at low pressures in addition to producing high-quality effluent [49]. Mortazavi et al. study outcome was the elimination of 85% fluoride and 98% hexavalent chromium using a nanofiltration membrane [66]. The findings of the study by Worou et al. on the removal of arsenic from water using nanofiltration membranes suggested that these membranes might eventually outperform all other arsenic removal technologies [46]. Additionally, Siddique et al. believed that nanofiltration is excellent in treating industrial effluent, softening water, and removing color as well as removing arsenic from water [3].

Saboori et al. conducted a study to evaluate the impact of various factors on the reverse osmosis system. According to the investigations, variations in the input solution's arsenic content had a substantial impact on the removal effectiveness of the metal, and among the tests on solutions with concentrations between 0.018 and 2 mg/L, the 1.5 mg/L solutions had the highest removal rate of 98% at the optimum pressure of 190 bar. Additionally, when pH rises, 5-valent arsenic particles break down from neutral to monoanionic, then to di-anionic form, increasing the proportion of arsenic elimination. By altering the solvent's viscosity, as well as by raising the osmotic pressure and the solvent and solute's permeability, the temperature rises also improved the effectiveness of removing arsenic from the solution. Temperatures between 20 and 30 °C were shown to have the greatest elimination of arsenic. This variable had little impact in the temperature range of 4–10 °C because of fluctuations in the solution's low viscosity. The ideal dosage of 1.5 mg/L at pH 9 and a temperature of 23 °C had been found to remove the most arsenic about 95.98% [18].

In a study, Golami et al. discovered that the reverse osmosis system performs best at 190 psi pressure, 25 °C temperature, and pH = 6.9 and attained a removal efficiency of over 99% [67]. In a study by Akin et al. the removal efficiency of arsenic from two reverse osmosis membranes (SWHR and BW30) was investigated. SWHR membrane outperformed BW30 in all test circumstances. The research's findings also showed that the pH has a significant impact on the removal of both kinds of 3 and 5-valent arsenic by the reverse osmosis membrane, with the removal of 5-valent arsenic being maximized at a pH above 4 and the removal of 3-valent arsenic at a pH over 9.1. The elimination of both forms of arsenic was enhanced when the pressure was raised. The removal of both forms of arsenic increased as the pressure in the reverse osmosis system, which functions as a driving force, increased, whereas variations in concentration had no impact on the effectiveness of the removal [68].

Mozafarian et al. elected the TFC-SR membrane after looking at five different types of reverse osmosis membranes, despite the PVD membrane having the greatest arsenic recovery (98.1%), because of its greater output flux than the PVD membrane (nearly twice) and removal efficiency (96.1%) [10]. The research done by Momtazan et al. to remove cadmium from drinking water using the reverse osmosis system indicated that this system is a highly favorable approach for doing so and can remove more than 98% of cadmium from drinking water. This research also demonstrated the usefulness of the reverse osmosis process in removing arsenic [69]. Chang et al. determined from their analysis of the removal of trivalent arsenic by low-pressure reverse osmosis and nanofiltration membranes that the removal of arsenic by both membranes is nearly constant in the pH range of 4–9, but dramatically rises at pH above 9. The removal efficiency of the reverse osmosis membrane did not change significantly as arsenic concentration increased (between 50 and 400 μg/L), but the removal efficiency of the nanofiltration membrane decreased by 10% and by 30% when Na_2_SO_4_ (0.1 Mm) salt was present. Due to the large size of the membrane pores, the nanofiltration membrane could only remove over 50% of the arsenic, but the reverse osmosis membrane could remove arsenic with an efficiency of over 90% [70].

In the study by Elcik et al. it was discovered that while pressure does not influence reverse osmosis membranes, it somewhat improves the removal efficiency in nanofiltration membranes. While reverse osmosis membranes do not significantly alter as a result of the oxidation of 3-valent to 5-valent arsenic, nanofiltration membranes are intended to increase removal efficacy. The effectiveness of reverse osmosis and nanofiltration membranes were both improved by the presence of hypochlorite. Reverse osmosis membranes removed 3-valent arsenic more effectively than nanofiltration membranes, and of the two nanofiltration membranes utilized, the NF90 membrane was more effective than the NF270 membrane, whereas two reverse osmosis membranes (XLE and BW30) were similarly effective [26].

Teimouri and Mahdiarfar conducted a study to assess safe arsenic removal technologies. Based on their findings, reverse osmosis and nanofiltration techniques were found to be the most effective membrane methods for removing arsenic from water. Additionally, it was observed that the reverse osmosis method can remove more contaminants at once whereas the nanofiltration procedure needs good-quality effluent [12]. Additionally, Kundu et al. discovered in a study on the removal of arsenic by membrane processes that all membrane processes had a greater capacity to remove 5-valent arsenic than 3-valent arsenic. The pH of the solution and the membrane's electric charge has a big impact on how much arsenic can be removed using membrane procedures. Reverse osmosis and nanofiltration are both efficient methods for removing arsenic, although in both cases, there is a risk of clogging from material deposition, which is minimized by the pre-treatment procedure [8]. Additionally, Rahidul Hassan et al. revealed that the best techniques for removing arsenic from polluted water are NF and RO [71]. The usage circumstances and effectiveness of various reverse osmosis and nanofiltration techniques are shown in Table 3.

**Table 3:** Comparing the efficacy of reverse osmosis and nanofiltration in the removal of arsenic from water.

Removal Process	Experiment conditions					Removal Efficiency (%)	Ref.
	Membrane type	Concentration	Temperature	Pressure	pH		
Nanofiltration	NF-300	100–382 μg/L	10–25 °C	310–724 kPa	1.2–8.8	As^5+^: 95	[65]
Nanofiltration	NF90-2540	100–1000 μg/L	27–37 °C	4–7 bar	3–11	As^3+^: 86.75–95.11	[60]
						As^5+^: 94.13–99.02	
Nanofiltration	NF90	20–100 μg/L	25 °C	138–552 kPa	4–10	As^3+^: 44	[64]
						As^5+^:89	
Nanofiltration	NF30	100–1000 μg/L	15–40 °C	2–12 bar	3.1–5	As^5+^: 77-88	[63]
Nanofiltration	NF90					As^5+^: 94.4–98	
Nanofiltration	NF	50–400 μg/L	20 °C	0.41–0.82 MPa	2–10	As^3+^: 10-40	[70]
Reverse osmosis	LPRO					As^3+^: 65-90	
Nanofiltration	NF90	100 μg/L	20 °C	3.1–5 bar	3.1–5	As^3+^: 67.72	[26]
Nanofiltration	NF270					As^3+^: 57.96	
Reverse osmosis	XLE					As^3+^: 98.23	
Reverse osmosis	BW30					As^3+^: 97.47	
Reverse osmosis	PVD	69.3 μg/L	8–21 °C	5–14 bar	5–9	As^3+^: 98.1	[10]
Reverse osmosis	TFC-SR					As^3+^: 96.1	
Reverse osmosis	FT30					As^3+^: 89.2	
Reverse osmosis	TFC-ULP					As^3+^: 83.2	
Reverse osmosis	BW30					As^3+^: 90	
Reverse osmosis	TE2521	0.0–2.5 mg/L	25–30 °C	190-210 psi	6–8	Total As: 95-99	[54]
Reverse osmosis	SWHR	50–750 μg/L	20 °C	10–35 bar	4.1–9.1	As^3+^: 92.5 As^5+^: 96.8	[68]
Reverse osmosis	TW40	0.2–18 mg/L	4–30 °C	190 bar	5–9	Total As: 95-99	[18]

## 6 Bioremediation and phytoremediation to remove arsenic from water

A new environmentally friendly method called bioremediation uses live microorganisms to eliminate environmental toxins like heavy metals [14]. Phytoremediation, which uses hardy plants to remove heavy metals including arsenic from soil and water, is one of the most significant bioremediation techniques. It is economical and environmentally benign. Many plants, such as Lemna Valdiviana, Pistia stratiotes, Eichhornia, Lepidium sativum, and Hydrilla verticillate, may acquire significant quantities of arsenic from polluted water. Despite its benefits, phytoremediation is not commercially available and requires considerable development [16,72,73].

## 7 Arsenic removal mechanism

One of the most crucial techniques for removing pollutants, such as arsenic, is the employment of membranes with a vast number of holes that, due to their selective nature, prevent some water elements from escaping. A driving force, such as the differential in pressure between the two sides, is necessary for this operation (feed and the permeate sides) [8,74]. A sophisticated high-pressure membrane technology called nanofiltration uses electrostatic charge repulsion and size exclusion to separate ions (Dona exclusion) [8,56]. A semi-permeable membrane is used to separate two solutions with various chemical densities in reverse osmosis, another high-pressure (5–120 bar) method of removing arsenic from water. The Ro process involves the direction of the water flux being reversed when the applied pressure is higher than the osmotic pressure [8,10].

## 8 Conclusion

Arsenic, being one of the most significant water contaminants, has long been a source of concern owing to its negative impact on human health and other living species. Membrane procedures are currently of great relevance among arsenic removal techniques because of their distinctive benefits. In this study, reverse osmosis and nanofiltration were explored as two membrane processes for the removal of arsenic. The findings indicate that these two processes are the most effective at removing arsenic among membrane processes, and their concentrations are up to below the standard limit. The elimination of arsenic's 5-valent form was carried out to a larger extent than that of its 3-valent form in each study that was investigated. Furthermore, compared to nanofiltration, the reverse osmosis technique was more efficient in eliminating 3-valent arsenic. Another finding from this investigation revealed that the average percentage of arsenite removed by the NF and RO membranes, respectively, was (10–95.11%) and (65–98.23%). Arsenate removal averaged between (77 and 99.02%) and (96.8%). In general, the operating settings for eliminating As^3+^ and As^5+^ species by nanofiltration were as follows: concentration 20–1000 μg/L, temperature 20–37 °C, pressure 1.38–8.2 bar, pH 2–11 and concentration 20–1000 μg/L, temperature 10–40 °C, pressure 1.38–12 bar and pH 1.2–11. While the reverse osmosis operating conditions for As^3+^ are 50–750 μg/L, temperature 8–21 °C, pressure 3.1–35 bar, and pH 2–10, the working conditions for As^5+^ are 50–750 μg/L, temperature 20 °C, pressure 10–35 bar, and pH was 4.1–9. It was found that increasing the pH considerably increased the performance of the membranes in all circumstances. The method's effectiveness was also impacted by the presence of various ions. The overall findings demonstrated that these two procedures, despite certain usage restrictions, have the potential to become the most effective methods of removing arsenic.

## Author contribution statement

All authors listed have significantly contributed to the development and the writing of this article.

## Funding statement

This research did not receive any specific grant from funding agencies in the public, commercial, or not-for-profit sectors.

## Data availability statement

Data included in article/supplementary material/referenced in article.

## Declaration of interest's statement

The authors declare no conflict of interest.
